# Ancient DNA and high-resolution chronometry reveal a long-term human role in the historical diversity and biogeography of the Bahamian hutia

**DOI:** 10.1038/s41598-020-58224-y

**Published:** 2020-01-28

**Authors:** Jessica A. Oswald, Julie M. Allen, Michelle J. LeFebvre, Brian J. Stucky, Ryan A. Folk, Nancy A. Albury, Gary S. Morgan, Robert P. Guralnick, David W. Steadman

**Affiliations:** 1 Florida Museum of Natural History, University of Florida, Gainesville, FL 32611 USA; 2 Biology Department, University of Nevada Reno, Reno, NV 89557 USA; 3 Illinois Natural History Survey, University of Illinois Urbana-Champaign, Urbana, IL 61801 USA; 4 Department of Biological Sciences, Mississippi State University, Starkville, MS 39762 USA; 5 National Museum of The Bahamas, Marsh Harbour, Abaco Bahamas; 6 New Mexico Museum of Natural History, 1801 Mountain Road NW, Albuquerque, NM 87104 USA

**Keywords:** Biogeography, Palaeoecology, Biodiversity

## Abstract

Quaternary paleontological and archaeological evidence often is crucial for uncovering the historical mechanisms shaping modern diversity and distributions. We take an interdisciplinary approach using multiple lines of evidence to understand how past human activity has shaped long-term animal diversity in an island system. Islands afford unique opportunities for such studies given their robust fossil and archaeological records. Herein, we examine the only non-volant terrestrial mammal endemic to the Bahamian Archipelago, the hutia *Geocapromys ingrahami*. This capromyine rodent once inhabited many islands but is now restricted to several small cays. Radiocarbon dated fossils indicate that hutias were present on the Great Bahama Bank islands before humans arrived at AD ~800–1000; all dates from other islands post-date human arrival. Using ancient DNA from a subset of these fossils, along with modern representatives of Bahamian hutia and related taxa, we develop a fossil-calibrated phylogeny. We found little genetic divergence among individuals from within either the northern or southern Bahamas but discovered a relatively deep North-South divergence (~750 ka). This result, combined with radiocarbon dating and archaeological evidence, reveals a pre-human biogeographic divergence, and an unexpected human role in shaping Bahamian hutia diversity and biogeography across islands.

## Introduction

The modern diversity and distribution of species are due to both natural factors across geological time, and human activities during the Quaternary. Humans have caused extinctions and contraction/fragmentation of ranges in countless species, while at the same time benefiting other species through introductions, habitat modification, and domestication. Long appreciated as places to study evolution, extinction, biogeography, and archaeology^1–3^, many islands also provide excellent settings to evaluate the relative roles of climate and associated habitat change versus human activities in shaping modern plant and animal communities e.g.^4,5^. In the West Indies, for example, the record of vertebrates from archaeological (cultural) and paleontological (non-cultural) sites reveals substantial extinction (species-level loss) and extirpation (population-level loss) of species since the late Quaternary. Some of these losses are thought to be related to the major changes in sea level, land area, and climate during the Pleistocene-Holocene Transition (the PHT; ~15,000 to 9,000 BP^6–9^). Even more extinction and extirpation of West Indian reptiles, birds, and mammals took place after human arrival ~7,000 BP^10–13^. For example, Cuba and Hispaniola were the world’s last refuge for ground sloths, which became extinct ~4,500 BP; their continental relatives, on the other hand, went extinct about 6,000 years earlier, at the end of the Pleistocene^4^. An endemic Jamaican monkey survived until only ~900 BP^12^.

**Figure 1:**
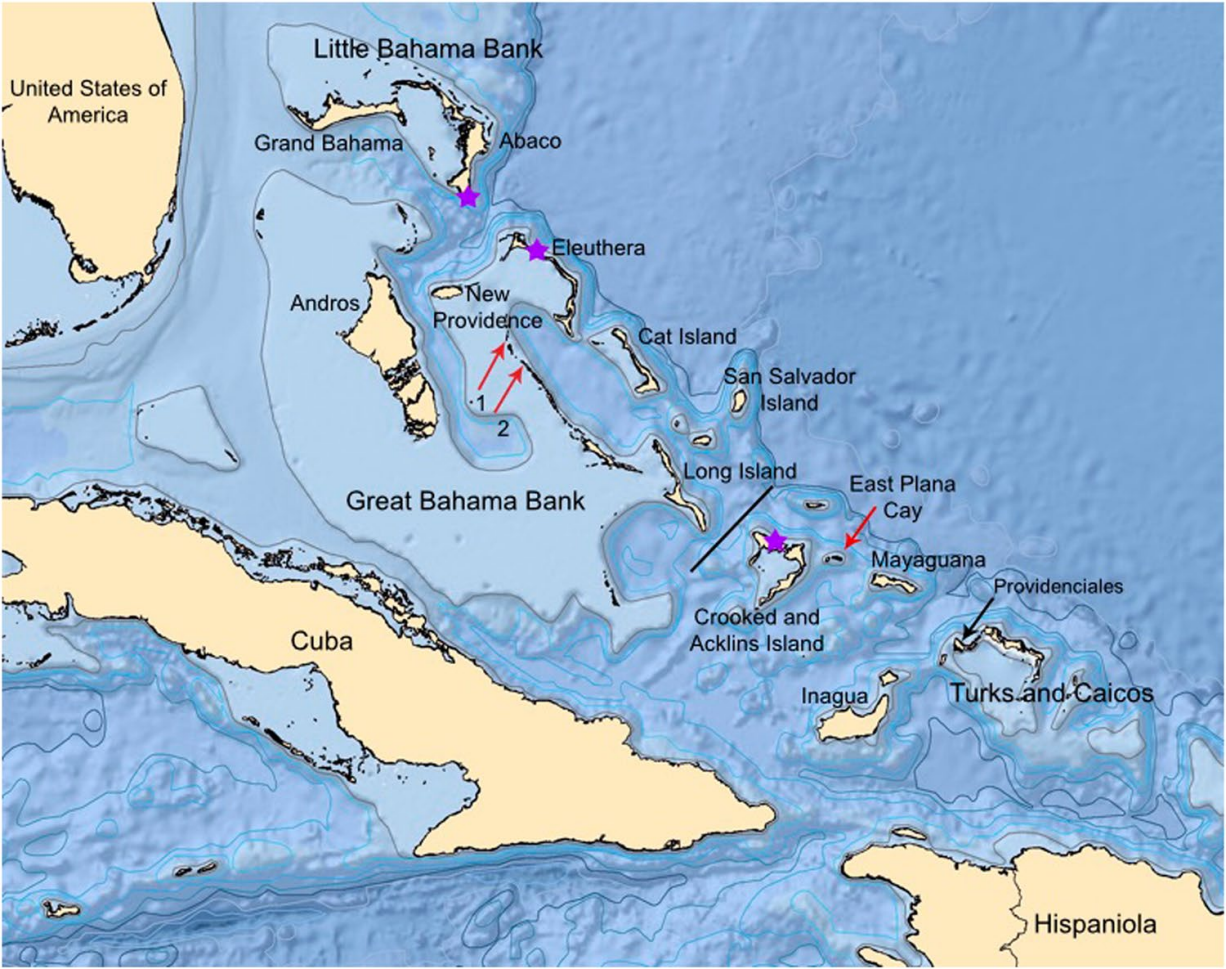
The Bahamian Archipelago. The lightest shade of blue represents the carbonate banks. On the Little Bahamas Bank, for example, are the islands of Grand Bahama and Abaco. Major islands on the Great Bahama Bank include Andros, New Providence, Eleuthera, and Long Island. The Crooked-Acklins Bank sustains Crooked and Acklins islands. Islands such as Mayaguana and Inagua were never connected to any other islands regardless of sea levels. The Lucayans colonized the Bahamas either from Cuba and/or from Hispaniola. Historically, Bahamian hutia occurred across the Bahamas. Today hutias are restricted to a few small islands (indicated by red arrows) including Little Wax Cay (1), Warderick Wells Cay (2), and East Plana Cay (labeled). Stars indicate the successful sampling localities for the ancient DNA portion of this study. The earliest archaeological site that shows hutia exploitation is from Major’s Landing on Crooked Island^42^, which is under the star on Crooked Island. The genetic break found in our data is indicated by the black bar between Long Island and Crooked and Acklins Islands (see Results). To make this figure, we cropped the bathymetry data layer from Eakins and Sharma^71^ to the Caribbean region using ArcMap version 10.1^72^.

The Bahamian Archipelago (The Bahamas + The Turks & Caicos Islands) extends from near Florida southeastward toward eastern Cuba and Hispaniola (modern day Haiti and Dominican Republic; Fig. 1). Islands of the Bahamian Archipelago lie atop shallow-water carbonate banks that are separated by deep water. While islands on a given bank were connected to each other during lowered sea levels of Quaternary glacial intervals, the banks themselves remained isolated from each other. The sea-level-controlled expansion and contraction of land area on these banks through Quaternary glacial-interglacial cycles^14^ has strongly influenced the archipelago’s terrestrial plant and animal communities^15^. During glacially lowered sea levels, Bahamian islands were not connected to North America or to neighboring Cuba and Hispaniola, although the Great Bahama Bank (GBB) was only 20 km away from Cuba during glacial intervals^7^ (Fig. 1). All colonization of the archipelago by terrestrial organisms therefore had to involve trans-oceanic dispersal. The geographic proximity of the Bahamian Archipelago and Cuba likely underlies their high number of shared extinct, extirpated, and extant species between these islands. Human colonization of the Bahamian Archipelago occurred fairly recently (AD ~800-1100), making it feasible to distinguish human impacts on biodiversity from those that occurred at the PHT.

The Bahamian Archipelago sustains only a single species of endemic non-volant mammal, the Bahamian hutia (*Geocapromys ingrahami*; Family Echimyidae, Subfamily Capromyinae^16^). The broader group of capromyine rodents are found in the Greater Antilles, Cayman Islands, Bahamas, and Swan Islands, although more than 50% of these species have been lost during the Holocene^17–20^. The oldest capromyine fossils are from the early Miocene of Cuba (*Zazamys veronicae*)^21^. Both modern representatives of this lineage (historically considered in the subfamily Isolobodontinae within the Capromyidae), *Isolobodon montanus* and *I*. *portoricensis*, became extinct during the Holocene^22,23^. Of the 13 extant hutia species (in 5 genera), nine are classified as vulnerable, endangered, or critically endangered (IUCN Red List of Threatened Species)^24,25^. Cuba is the modern and historical center of diversity of the family with 10 extant species found on the mainland and offshore islands^26,27^. Recent extinctions, since European settlement, include *Geocapromys columbianus* (from Cuba) and *G*. *caymanensis* (from the Cayman Islands). Based on morphology, these two extinct species are closely related to the Bahamian hutia^27^. Additional recent hutia extinctions include *Capromys pilorides lewisi*, an endemic subspecies from the Cayman Islands, and *G. thoracatus* from the Swan Islands^28–30^. The only extant species of hutias outside of Cuba are the endangered *Plagiodontia aedium* from Hispaniola and two vulnerable species of *Geocapromys*, *G. brownii* endemic to Jamaica, and *G. ingrahami*, the subject of this paper.

*Geocapromys ingrahami* is restricted today to three small Bahamian islands: East Plana Cay (Plana Cays) and Little Wax Cay and Warderick Wells (both in the Exuma Cays; Fig. 1). Only the East Plana Cay population is regarded as indigenous; hutias from East Plana Cay were transported to Little Wax Cay in the 1970s and to Warderick Wells in the 1980s^24,31^. The three modern populations of Bahamian hutias represent the nominate subspecies of *Geocapromys ingrahami*, described by Allen^32^ from East Plana Cay. However, Bahamian hutias were distributed much more broadly in the past and are abundant as fossils, especially on the islands of the Great Bahama Bank (GBB; Fig. 1) and in association with species that went extinct before human arrival on the islands, such as the owl *Tyto pollens*^29,33,34^. Morphological variation in cranial characters from these extirpated populations led Lawrence^35^ to describe two new subspecies of *G. ingrahami*, *G. i. abaconis* from Abaco (on the Little Bahama Bank, or LBB; Fig. 1), and *G. i. irrectus* from Eleuthera, Exuma, Long Island, and Crooked Island. Allen^36^ identified a large sample of hutia fossils from Exuma (Little Exuma) as *G. i. irrectus*, although this variation in morphology could be driven by resource availability^37,38^.

The population biology of Bahamian hutia was dramatically altered by the arrival of the Lucayan people to the Bahamian islands, which based on archaeological evidence, occurred from Cuba or Hispaniola^39,40^ as early as the eighth or ninth centuries AD. Early human settlements have been documented on Eleuthera, New Providence, Abaco, and San Salvador^40,41^. Zooarchaeological, morphometric, and isotopic studies of archaeological hutia specimens demonstrate that Lucayans exploited hutias for food and had significant impact on some hutia populations in terms of their geographic distribution, diet, and possibly body size^42^. Bahamian hutia bones have been found from archaeological sites on eight islands spanning the Little Bahama Bank (LBB; Abaco + Grand Bahama), GBB, Crooked-Acklins Bank, and the Turks & Caicos Islands (TCI)^43^ (Fig. 1). While hutias may have been exploited throughout Lucayan occupation of the archipelago, lack of directly dated hutia specimens from archaeological contexts has limited, until now, our understanding of human use throughout the region. To date, the earliest direct radiocarbon dates on archaeological hutia bones are cal AD 1330–1440 at Major’s Landing on Crooked Island (Fig. 1). This exploitation extended into the period of European exploration (with the youngest dated material also from Crooked Island; AD 1450 to 1620), when hutias disappeared across nearly all Bahamian islands^43,44^.

On banks outside of the GBB, the long-term, natural occurrence of *G*. *ingrahami* has been called into question. On Abaco (LBB), for example, hutia fossils do not occur in the very rich Sawmill Sink site prior to human arrival on the island at AD ~1000. Hutia bones are present on Abaco, however, after humans colonized the island^6,45^. Extensive radiocarbon dating of hutia material from other banks (e.g., Crooked, Mayaguana) has been limited prior to this work. A bone-rich site on Middle Caicos Island (TCI) also lacks hutias, regardless of time interval (DWS pers. obs.). This suggests that, just as on Abaco, hutias were likely not native to this region^24^. An ongoing study of a large hutia assemblage from the Palmetto Junction archaeological site on Providenciales (Caicos Bank) indicates that Bahamian hutias were human-translocated to the TCI by at least the early 15th century (Supplementary Table S1; see also^43^). The archaeological specimens from Palmetto Junction are larger than modern specimens from East Plana Cay; isotopic data from these specimens suggest that some individuals consumed ^13^C-enriched foods that were available through human supplementation or opportunistic foraging; this is a finding shared with archaeological specimens from Crooked Island (e.g., Major’s Landing site)^42^. Taken together, it seems likely that the Lucayans were actively managing some Bahamian hutia populations by AD 1400 or earlier.

**Table 1:** Data for *Geocapromys ingrahami* ancient DNA samples.

UF Number	Beta-Number	Sample Name	Protocol	% mt coverage	Number of reads	Min. read depth	Max. read depth	Mean read depth	SRA Number (PRJNA578925)	GenBank Number
322961	502523	Abaco 1	shotgun	99.6%	7,272	0	137	34.7	SAMN13192402	MN695892
322959	520445	Abaco 2	enrichment	100%	361,147	298	13,342	2,821.9	SAMN13192403	MN695894
322948	520443	Eleuthera 1	shotgun	99.0%	2,045	0	27	9.9	SAMN13192404	MN695896
322947	520442	Eleuthera 2	enrichment	99.5%	237,460	0	15,097	1,705.1	SAMN13192405	MN695897
322960	502524	Crooked Island 1	shotgun	98.9%	2,436	0	73	11.7	SAMN13192406	MN695893
322949	520444	Crooked Island 2	enrichment	100%	292,680	76	12,951	2,481.6	SAMN13192407	MN695895

Percent coverage is based on the reference sequence of *G*. *brownii* (KU 892767.1), which is 16,566 bp in length.

Previous work documents a link between people and the biogeography of Bahamian hutias, beginning with human settlement of the Bahama archipelago by AD ~800–1000 and continuing into modern times^32,37,38,42,46–49^. Nevertheless, significant gaps remain in our knowledge about how humans have affected the genetic diversity and distribution of Bahamian hutias through time. In this paper we combine paleontology (fossil records, chronometric data), archaeology (cultural context), and phylogenetics (ancient DNA) to explain changing historical diversity of Bahamian hutias.

We address questions about genetic diversity through time and human use of indigenous animals in the Bahamian Archipelago: To what extent did hutias disperse across the island group by natural means (e.g., rafting or swimming) vs. human transport? If the inter-bank distribution of Bahamian hutias was due to natural dispersal (rafting, swimming) between islands, unaided by humans, such dispersal most likely took place during lowered sea levels of the Pleistocene, no later than ~10,000 BP during the archipelago’s last full exposure of land (when Cuba and the GBB were ~20 km apart). Therefore, hutia fossils should be present in pre-cultural contexts (>1,000 BP) on any bank in the archipelago. Accordingly, genetic data on hutias should be geographically structured to reflect inter-bank differences. Alternatively, if humans transported hutias to islands outside of the GBB within the past 1,000 years, all radiocarbon dated hutia bones outside of the GBB should fall within the period of human presence in the islands, even those from non-cultural strata, and inter-bank genetic differentiation should be minimal.

## Results

### Radiocarbon dates

**Figure 2:**
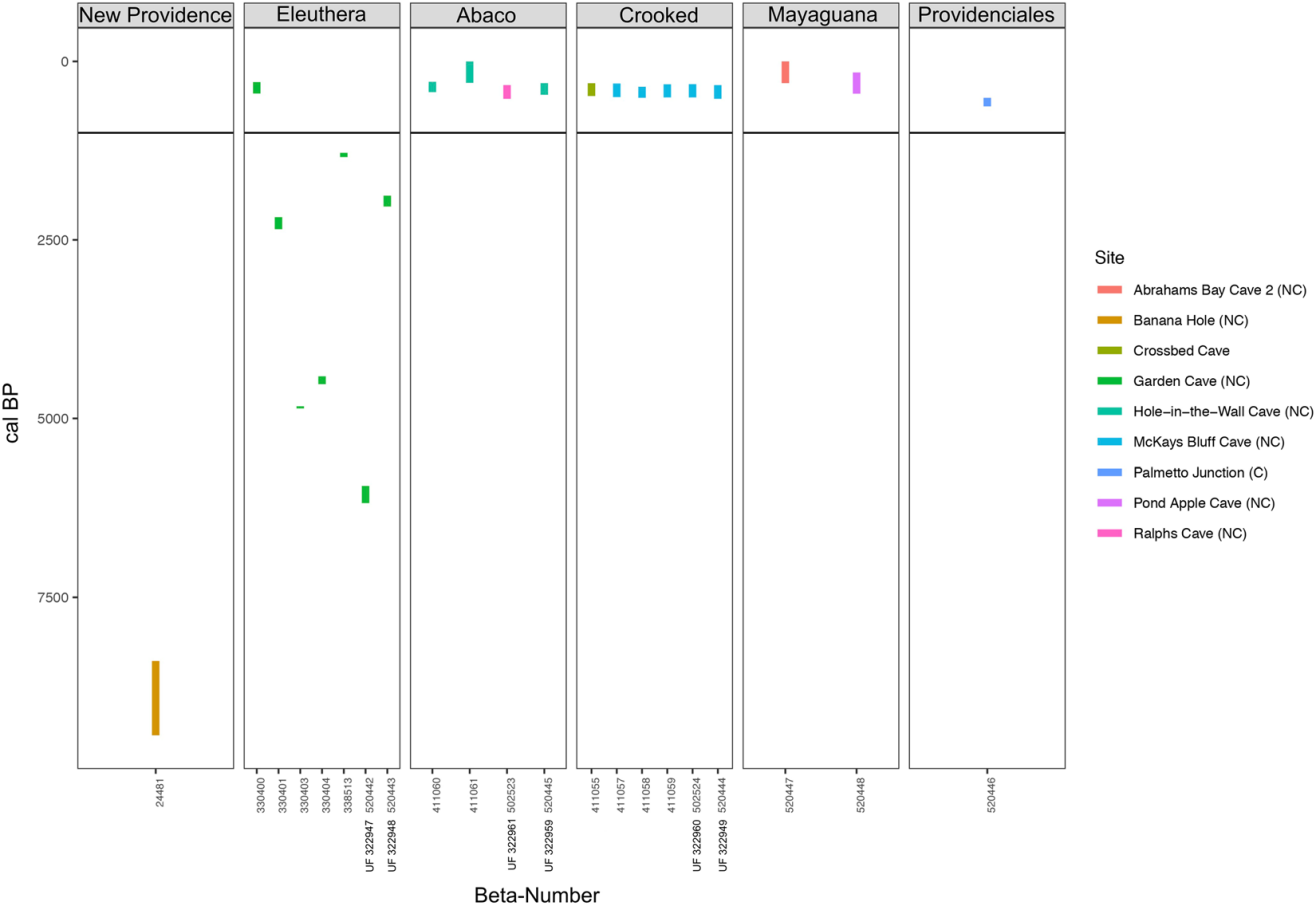
Plotted radiocarbon dates (cal BP) for individual bones of hutia (*Geocapromys ingrahami*) from the Bahamian Archipelago. UF numbers for the aDNA samples are next to the beta numbers. The length of the bar corresponds to the minimum and maximum dates for each measurement. Eleuthera and New Providence are on the GBB, Abaco is on the LBB, Crooked Island is on the Crooked-Acklins Bank, Mayaguana was isolated through time, Providenciales is on the Caicos Bank (see Fig. 1). The black line at approximately 1,000 years BP marks the time of colonization of the Bahamas Archipelago by the Lucayans. See Supplementary Table S1 for radiocarbon (^14^C) and stable isotope (δ^13^C, δ^15^N) data and date conversions for all samples.

Twenty one of the 29 *Geocapromys ingrahami* specimens (all from the GBB) yielded collagen and therefore an AMS ^14^C date (Fig. 2, Supplementary Table S1). The GBB hutias without collagen from New Providence and Long Island were associated with species of birds that are characteristic of Pleistocene rather than Holocene faunas^9,44,45^; therefore, their lack of collagen is likely due to their antiquity. The oldest hutia fossils with collagen also are from the GBB. These fossils date to the early Holocene with the oldest radiocarbon date from New Providence at 9,432–8,392 cal BP (Fig. 2; Supplementary Table S1). *Geocapromys ingrahami* fossils from the LBB (Abaco) all post-date human arrival. The same is true for the Crooked-Acklins Bank, Mayaguana Bank, and Caicos Bank. The youngest dated hutia fossils, from Mayaguana and Abaco, are no more than several centuries old. Samples where aDNA was sequenced ranged from as old as ~6,000 cal BP (Eleuthera; GBB) to as young as cal AD ~1400 to 1600 (Abaco, Crooked; Supplementary Table S1). The samples from Mayaguana and Providenciales that did not yield aDNA also were only several centuries old. The δ^13^C and δ^15^N values from all samples fall within expected values for terrestrial herbivores with C3-dominated diets.

### Sequence quality and coverage

The samples enriched for mitochondrial data generally recovered two orders of magnitude more on-target reads than shotgun-sequenced samples, regardless of the sample’s age (Table 1). All samples recovered at least 99% of the reference genome. The enriched sample from Abaco (~320 years old; UF 322959) recovered the highest number of reads overall (361,147) with a minimum read depth of 298 and maximum of 13,342. The shotgun-sequenced sample from Eleuthera (~2,000 years old; UF 322948) recovered the fewest on-target reads (2,045) with a maximum sequence depth of 27 reads. An even older sample from Eleuthera (~5,270 years old; UF 322947) that we enriched recovered 237,460 on-target reads, indicating that enrichment success was not clearly related to sample age.

### MapDamage

The most common form of DNA damage found in aDNA is elevated C-T transitions rate occurring on the 5′ end^50,51^. This pattern can be used as evidence that the DNA reads in our dataset are targeted aDNAs and not the result of modern DNA contamination. The DNA damage patterns for the mapped gene regions of all hutia ancient samples are consistent with those of ancient DNA (Supplementary Fig. S1).

### Phylogeny and divergence estimation

**Figure 3:**
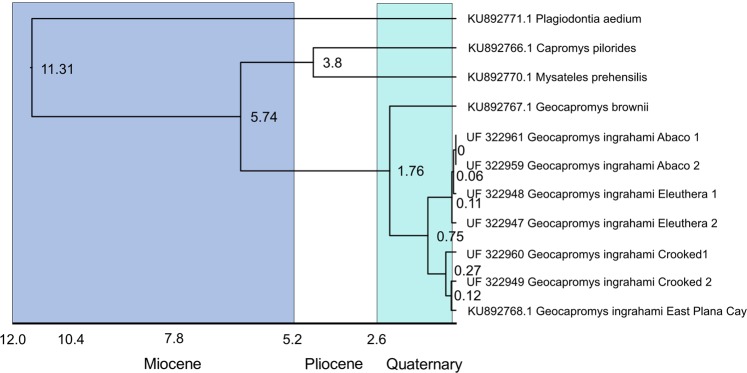
Fossil calibrated phylogeny of Capromyinae based on mitochondrial genome data. Please note that this is a subsection of the entire phylogeny included in analyses. See Supplementary Fig. S2 for maximum likelihood phylogeny, Supplementary Fig. S3 for the 95% highest posterior density divergence time, and Supplementary Fig. S4 for posterior probabilities. Divergence times (in millions of years) are given at the nodes. The Miocene Epoch (23-5.2 Ma) is purple. The Pliocene Epoch (5.3-2.6 Ma) is white. The Pleistocene Epoch 2.6 Ma to ~12,000 years ago is light blue. The divergence time between the Abaco (LBB) + Eleuthera (GBB) and the Crooked Island (CAB) + East Plana Cay (extant population) is 750,000 years. All posterior probabilities are 1 for these taxa, see Supplementary Fig. S4].

The RAxML phylogeny recovered *Geocapromys brownii* as sister to *G*. *ingrahami*. We found two well-supported clades within *G*. *ingrahami*. One consists of the ancient samples from Abaco (LBB) and Eleuthera (GBB), whereas the other consists of ancient samples from Crooked Island (Crooked-Acklins Bank) and the modern sample from East Plana Cay (Supplementary Fig. S2). The fossil calibrated phylogeny produced by BEAST2 recovered the same topology as in the RAxML phylogeny except for the placement of *Carterodon sulcidens* due to the constrained topology in the BEAST2 analyses (Supplementary Figs. S2–S4). The divergence time between the Capromyinae and the MRCA node of *Carterodon sulcidens* is 19.14 to 16.68 Ma (95% highest posterior density; Supplementary Fig. S3). This divergence is compatible with the divergence time for that same node (18.2–14.8 Ma) and sister taxa for those analyses recovered by Fabre *et al*.^52^ (Supplementary Figs. S3 and S4). The fossil used to calibrate this node, *Zazamys veronicae*, is a stem fossil and therefore the most recent common ancestor of the Carterodoninae + Capromyinae is likely older. The estimated divergence between the northern (Abaco-Eleuthera) and southern (Crooked Island – East Plana Cay) samples of *G. ingrahami* occurred during the Pleistocene between 1.04 to 0.52 Ma (Fig. 3, Supplementary Fig. S3). Within the Capromyinae the posterior probabilities for the topology are all 1 (Supplementary Fig. S4).

### Pairwise distance

The highest nucleotide divergence was between the northern (Eleuthera-Abaco) and southern (Crooked-East Plana Cay) samples of *G*. *ingrahami*. The samples from Abaco (LBB) and Eleuthera (GBB) were an average of 2.2% divergent from the Crooked Island and East Plana Cay (modern) samples. The Abaco and Eleuthera samples were 0.2–0.3% divergent from each other. The Crooked Island and East Plana Cay samples were 0.4–0.9% divergent from each other (Supplementary Table S2).

## Discussion

Combining Quaternary paleontological and archaeological evidence is important to understand the history of contemporary biodiversity and species distributions e.g.^53^. The aim of our study was to determine the role of natural processes (e.g., rafting) versus human activities in shaping past diversity and distribution of the single non-volant mammal species found in the Bahamas, *G. ingrahami*. We used radiocarbon dates and ancient DNA to understand the phylogenetic diversity and the historical distribution of this hutia species. Our chronometric and genetic data reveal a far more long-term and active human role in Bahamian hutia historical diversity and biogeography than previously recognized.

Both radiocarbon and ancient DNA evidence supports the hypothesis that people transported hutias across much of the Bahamian Archipelago. Radiocarbon dates indicate that none of the hutia fossils outside of the GBB are older than cal AD 1300–1400, which is several centuries after Lucayan arrival in the Bahamas. While individual hutias may have naturally dispersed across the Bahamian Archipelago ~700 years ago, the most parsimonious explanation, given evidence of human exploitation of this species, is that humans moved them across the islands. Because of the nature of the fossil record, it remains possible that pre-human hutia fossils from outside of the GBB will be found. However, we doubt that the lack of hutia fossils prior to human arrival on non-GBB islands is a sampling artifact because two of these islands (Abaco, Middle Caicos) are also home to rich paleontological sites that have yielded tens of thousands of vertebrate fossils^44^. The minimal sequence divergence between individuals from Eleuthera (GBB) and Abaco (LBB) also supports a prehistoric human transport of Bahamian hutia from the GBB to the LBB, a distance of 90 km today (Figs. 1 and 3). Considering that we lack fossil evidence that Bahamian hutias were native to Crooked Island, the 2% divergence between the LBB + GBB versus the Crooked Island (Crooked-Acklins Bank) samples of hutias could be driven by a number of factors that will require additional samples and genetic data to more fully understand. One hypothesis is that the southern GBB was home to a divergent population (not yet sampled in this study) that was transported to Crooked Island or is more closely related to an extinct taxon of *Geocapromys* from elsewhere, e.g., Cuba.

Our genetic results show that Bahamian hutia, now restricted to a few small cays, is less genetically diverse today than it was just several hundred years ago when it occurred nearly across The Bahamas. In particular, a genetically distinct population that previously lived in Abaco and Eleuthera is now extinct. Based on morphology, Lawrence^35^ described two subspecies of *Geocapromys ingrahami,* with the northern subspecies, *G. ingrahami abaconis*, restricted to Abaco. We now know that hutias from Abaco (LBB) and Eleuthera (GBB) show negligible genetic divergence and that the genetic diversity represented by these populations is lost. Possible morphological variation between the populations needs to be re-evaluated using the much larger samples now available through recent excavations. The other subspecies described based on morphology by Lawrence^35^ was *G. i. irrectus* from Crooked Island and the GBB islands of Eleuthera, Exuma, and Long Island. Given the new genetic data, we question the monophyly of *G. i. irrectus*. The extant Bahamian hutia populations (on East Plana Cay, Little Wax Cay, and Warderick Wells) do not represent the full genetic diversity present in the Bahamas from 500 to 350 years ago when hutias also lived on other islands and banks. Furthermore, hutias on East Plana Cay, traditionally considered to be the only surviving indigenous population, may instead be the product of earlier human transport, as the modern population shows little divergence from the population that was once on Crooked island. Regardless of origin, the long-term future of this vulnerable species depends on the survival of populations on small cays uninhabited by people.

Both historically and today, the Cuban and Bahamian faunas are highly similar. Among the many examples of shared species are the parrot *Amazona leucocephala*, hummingbird *Chlorostilbon ricordii*, and warbler *Setophaga pityophila*. When late Quaternary fossils and extinct/extirpated species are considered, the similarities expand to include such species as a crocodile (*Crocodylus rhombifer*), caracara (*Caracara creightoni*), barn owl (*Tyto pollens*), woodpecker (*Xiphidiopicus percussus*), kingbird (*Tyrannus cubensis*), and others. Cuba is the center of extinct and extant capromyine diversity, whether for *Capromys* or *Geocapromys*^26,27^. Morphological data suggest that *G. ingrahami* and two extinct species, *G. columbianus* from Cuba and *G. caymanensis* from Cayman Brac and Grand Cayman, form a group within *Geocapromys*, distinct from *G. brownii* of Jamaica and the recently extinct Swan Island hutia *G. thoracatus*^27,54^. It is likely that the most recent common ancestor between Cuban *Geocapromys* sp. and *G*. *ingrahami* rafted to the GBB from Cuba during a Pleistocene glacial interval when the GBB was only 20 km from Cuba. The estimated genetic divergence between *G*. *brownii* and the Bahamian hutia is ~1.8 million years (early Pleistocene). The eventual inclusion of ancient DNA from fossil Cuban *Geocapromys* samples may fill in the phylogenetic gaps between these two spatially disparate representatives of this genus.

Previous archaeological evidence across the Bahamian Archipelago indicates that by the 15^th^ century and perhaps earlier, Lucayans translocated and possibly managed some Bahamian hutia populations^42^. The chronological correlation between Lucayan settlement of the region and the presence of hutias suggests that the pre-Columbian and early historical-era (post-1492) distribution of hutias was in part due to intentional human activity and successful management of some hutia populations. Further corroborating evidence comes from ancient DNA showing no inter-bank genetic differentiation in the northern Bahamas. The mutualistic relationship between pre-Columbian people and Bahamian hutias collapsed, however, with European colonization. The demise of the Lucayans, historic-era landscape changes, and the introduction of Old World mammals such as rats (*Rattus* sp.), mice (*Mus* sp.), and cats (*Felis catus*) decimated hutia populations across the island group.

Our work provides a basis for next-step studies to clarify the dispersal history and genetic diversity of Bahamian hutias, and the scale of long-term anthropogenic impact on each. Continued emphasis on developing a rigorous absolute chronology across paleontological and archaeological sites is essential to link temporal patterns in hutia dispersal, extirpation, persistence, and human activities. The relatively deep divergence between northern and southern populations of Bahamian hutia in our genetic data is striking. At this time, we do not know if there were populations with intermediate divergence across this north – south gradient within the GBB, or the relationships between extinct *Geocapromys* taxa from Cuba and Bahamian hutia. Additionally, more genetic and chronometric data from archaeological contexts and from Holocene paleontological deposits from minor banks will refine what we know about the movement of Bahamian hutias by people during the past millennium. In combination with more genetic data, the morphological variation found in this species among paleontological and archaeological specimens from different islands and banks needs to be investigated, especially to separate signatures of potential human selection from adaptation or morphological drift due to population isolation. Finally, the eventual inclusion of genomic data from *Geocapromys* samples from Cuba, the Cayman Islands, and Little Swan Island will provide a much clearer understanding of both the phylogenetic relationships among the five currently recognized species in this genus and the broader biogeographic history.

## Materials and Methods

### Radiocarbon dating

We selected 29 *Geocapromys ingrahami* specimens (28 fossil, 1 archaeological) from the Florida Museum of Natural History (UF) collections that represent seven islands and five banks (Supplementary Table S1). All radiocarbon (^14^C) dating and isotopic measurements took place at Beta Analytic Inc. The calibration data set follows IntCal13. Each age determination is an accelerator-mass spectrometer (AMS) ^14^C date on purified collagen from an individual hutia bone of known provenience. For each site, when possible, we chose bones from both the upper (presumably younger) and lower (presumably older) parts of the sedimentary sequence. For details on the methods of AMS ^14^C dating, including pretreatment of the bones, see https://www.radiocarbon.com/radiocarbon-dating-p.htm.

### aDNA sample information

We chose eight fossils and one archaeological specimen from five Bahamian Banks for ancient DNA extraction and analysis. Our six successfully extracted samples represent two bones each from the Great Bahama Bank (Eleuthera), Little Bahama Bank (Abaco), and the Crooked-Acklins Bank (Crooked Island; Fig. 1, Supplementary Table S1). The two fossil specimens from the Mayaguana Bank and the archaeological specimen from the Caicos Bank had inadequate DNA. A part of each extracted bone also was radiocarbon dated (Supplementary Table S1). On each island, we performed shotgun sequencing on one sample, and then performed targeted enrichment and sequencing second sample for the same island (see below).

### aDNA sample preparation

DNA extractions were performed in a UF lab dedicated exclusively to extracting ancient DNA. Each sample was processed on a separate day and each extraction had negative controls. We modified the methods of Yang *et al*.^55,56^ for DNA extraction from bone (following^57^). Briefly, the samples were soaked in a 5% bleach solution to remove surface contaminants. A piece of the shaft (diaphysis) weighing ~500 mg was removed from the fossil, crushed in liquid nitrogen, and then combined with 949 ul of 0.5 M ethylenediaminetetraacetic acid, 25 ul 20 mg/ml proteinase K, 21 ul of 10 mg/ml 1,4-dithiothreitol (DTT), and 5 ul of 10% sodium dodecyl sulfate (SDS). Samples were incubated at 60 °C for 24 hours and intermittently vortexed. Samples then were concentrated with an Amicon Ultra-4 Centrifugal Filter Unit, purified using a Qiagen QiaQuick MinElute Kit, and eluted in 48ul of EB buffer. We quantified the DNA extractions with a Qubit 2.0 Fluorometer and Agilent 2100 Bioanalyzer to determine fragment size and yield.

### Library preparation, shotgun sequencing, and sample enrichment

Library preparation was performed with a Swift Biosciences Accel-NGS Methyl-Seq DNA library kit following the standard kit protocol but excluding the bisulfite conversion step. This kit uses a uracil-tolerant polymerase and performs well with ancient samples (Swiftbiosci.com). The bead-to-sample ratios for the SPRI bead clean-up steps were modified for the ancient sample libraries to capture more small fragments relative to the modern samples (post-extension SPRI ratio 1.8; post-ligation SPRI ratio 1.6; post-PCR SPRI ratio 1.6). We used 14 PCR cycles during the amplification steps. Library concentration and fragment size were determined with a Qubit 2.0 Fluorometer and Agilent 2100 Bioanalyzer, respectively. At this step we sent three samples for whole genome shotgun sequencing (lllumina (2500) 100 bp paired-end sequencing) at Roy J. Carver Biotechnology Center/W. M. Keck Center, University of Illinois at Urbana-Champaign, where samples were cleaned, quantified via qPCR, and pooled for sequencing. Once we determined that the samples were yielding high-quality mitochondrial sequences, we changed to a target enrichment protocol with probes targeting the mitochondrial genome (described below) to reduce costs and increase on-target reads on the remaining three samples.

### Enrichment

A custom biotinylated RNA bait set (80mer probes with 4x tiling; 819 total probes) for hutia mitochondria was designed and synthesized by Arbor Biosciences (Ann Arbor, Michigan) based on *Geocapromys brownii* (KU 892767.1), the most complete and annotated mitochondrial genome closely related to *G*. *ingrahami* available on GenBank. To maximize mitochondrial read coverage and reduce high levels of exogenous environmental DNAs commonly found in aDNA samples, we performed two target capture reactions with this bait set, using the post-enrichment product of the first reaction concentrated with SPRI magnetic beads in a bead:sample ratio of 2X as input for the second reaction. Each target capture followed the myBaits protocol v. 4 with these modifications: for sequence capture, a hybridization temperature of 60 °C for 48 hr; for post-capture enrichment, an annealing temperature of 60 °C and 15 total cycles; for final post-enrichment cleanup, a bead:sample volume ratio of 1.8 × . Libraries were not multiplexed for sequence capture following manufacturer recommendations for aDNA; they also were not concentrated prior to capture, and ranged from < 1 to 3.8 ng total input. For the amplification step of the second round of target capture, we split the sample into two tubes for PCR (of 15 cycles) with a volume of 25 uL per reaction tube (i.e. 12 total PCRs for 6 samples). This was intended as a precaution against stochastic PCR amplification bias that can result from reactions with high cycle numbers. After amplification, the products of the two tubes for each sample were pooled for further processing. The post-capture libraries were cleaned, pooled, and sequenced on an Illumina MiSeq. 2 × 150nt PE Nano v2 at Roy J. Carver Biotechnology Center/W. M. Keck Center, University of Illinois at Urbana-Champaign.

### DNA read clean up

We first screened for significant irregularities in reads using FastQC v0.10.1 (Babraham Bioinformatics; following^57^). Adaptors were identified and trimmed using (fastx_clipper), followed by two additional adapter trimming steps due to additional adapter sequence added in the Swift Biosciences kit (Swift personal communication)^57^. The Swift kit adds a low complexity polynucleotide tail to the 3′ end that can also be observed at the beginning of read 2 given sufficiently short insert sizes (Swift personal communication)^57^. All forward reads were mapped to the reference genome (*Geocapromys brownii*, KU892767.1) using Geneious (v. 11.1.4) iterative reference-based assembler, and manually examined to determine the length of nucleotides (nt) that needed to be trimmed from the 3′ end, visible as mismatching GA or TC tails in read alignments (following^57^). Separately, all reverse reads also were mapped to the reference mitochondrial genome^57^. For each sample, 15 base pairs were trimmed from the 3′ end of read 1 (custom script) and also from the 5′ end of read 2 using fastx_trimmer (following^57^). Next, each sequence read was quality trimmed (fastq_quality_trimmer) from the 3′ end to remove bases with a Phred score less than 28 using a sliding window of 1nt; any reads fewer than 20nt in length were removed in the ancient samples^57^.

### Locus assembly

With the resultant reads, we used the Map to Reference feature in Geneious (v.11.1.4) with five iterations to map our reads to *Geocapromys brownii* (KU 892767.1). For the shotgun sequences, the sensitivity was set to high to avoid incorporating non-target reads getting incorporated into the assembly. Sequence data pile ups were evaluated by eye to identify possible sites with deamination and degradation. Single nucleotide polymorphisms (SNP) between the reference and the ancient hutia reads were further evaluated by eye to determine how many sequences supported the SNP difference and whether it was ambiguous, or if it was a site with deamination or degradation. Ambiguous sites (mostly in the D-Loop that were 50-50 SNP calls) were coded as “N” to be conservative. Consensus sequences were then extracted and used in the phylogenetic analysis. Finally, we assessed the DNA damage patterns for all samples using MapDamage^58^.

### Phylogenetics

To place *Geocapromys ingrahami* individuals in a phylogeny, we gathered mitochondrial genome data for species in the Echimyidae, Octodontidae, and Ctenomyidae on GenBank (Supplementary Table S3). The *Geocapromys ingrahami* mitochondrial sequence data on GenBank (KU892768.1) are from USNM 395696 (collected November 1969). This specimen lacks locality information. In the 20^th^ century, *G*. *ingrahami* was restricted to East Plana Cay until individuals were introduced to other small cays post the 1970s^24,31^. Accordingly, herein, we refer to this individual as being from East Plana Cay. *Chinchilla lanigera* was used as an outgroup. We used MAFFT to align the mitochondrial sequences (total base pairs: 17,599) and used GBlocks^59^ to remove sites that were poorly aligned or with substantial missing data (mostly within the D-Loop; resulting in 15,161 base pairs of data; alignment provided as electronic Supplementary Material). We used jModelTest-2.1.10^60,61^ and five substitution schemes to determine the best substitution model for the data. We chose the top three models based on AIC values (Supplementary Table S4). We performed phylogenetic analyses in RAxML (8.2.11)^62^ with the GBlocks^59^ trimmed mt genome dataset under a GTR + Γ + I model. The three partitions were protein-coding sequences (CDS), rRNA and tRNA sequences, and non-coding sequences; 10,000 bootstrap replicates (option “-f a”) were used to assess phylogenetic support.

### Divergence time estimation

We used BEAST 2.6.0^63^ and three fossil calibrations for phylogenetic divergence dating with the following settings: a GTR + Γ + I site model with 4 gamma count categories, a shape of 0.675, and proportion of invariant sites of 0.448 both of which were also set to be estimated during analyses. The latter two settings (shape and proportion of invariant sites) are produced during the jModelTest analysis. Additional settings included a relaxed lognormal clock model set to estimate a range of values between 0.0001–0.1 with an initial setting of 0.015 (based on a 3% clock for the molecular clock rate for rodents between 2–4% per million years^64^); a calibrated yule model for the tree prior; and a birthrate prior set as gamma with an alpha value of 0.001 and a beta value of 1000. The three fossils used to calibrate the phylogeny were set with a log normal distribution. First, we used the Early Miocene (18.8-16.3 my) hutia *Zazamys veronicae* from Cuba^65^ as a calibration date. Based on tooth morphology, *Zazamys veronicae* was a representative of the subfamily Isolobodontinae within the Capromyidae (when the hutias were considered their own family)^65^. We placed this fossil on the node of the clade containing Capromyinae and the sister subfamily Carterodontinae with the following settings: Mean = 0, SD 0.7, Offset = 16.56. With *Z*. *veronica*e at this node we constrained the topology following the results of Fabre *et al*.^52^ and Courcelle *et al*.^16^ that recovered *Carterodon sulcidens* sister to the Capromyinae. Mitochondrial data alone does not recover this sister relationship between Capromyinae and Carterodontinae (RAxML phylogeny herein; see^52^). Following Fabre *et al*.^66^, we used the Late Miocene (10.9-6.0 my) *Pampamys emmonsae*^67,68^ to calibrate the node of *Hoplomys*, *Myocastor*, *Proechimys*, and *Thrichomys* (Mean = 0, SD = 1.0, Offset = 5.81). Further following Fabre *et al*.^66^, we used *Xenodontomys simpsoni*, considered a crown ctenomyid from the late Miocene (10.9-5.7 my), as a calibration on the node of the clade containing the Octodontidae + Ctenomyidae (Mean = 0, SD = 1.0, Offset = 5.51). We ran the BEAST2 analysis twice, first with a chain length of 50 million generations with second analysis with an additional 25.5 million generations to reach acceptable effective sample size (ESS) values. We discarded 10% of burn-in and determined parameter convergence in Tracer^69^. To determine pairwise genetic distance across *Geocapromys ingrahami* samples, we used the R package Pegas^70^ with the GBlocks^59^ trimmed mitochondrial genome datasets. We used pairwise deletion to ignore sites with missing data.

## Data availability

Raw sequence data can be found on NCBI Sequence Read Archive (SRA) PRJNA578925 (SAMN13192402-SAMN13192407; see Table 1). Mitochondrial genome data is also available on GenBank (MN695892-MN695897; see Table 1). The GBlocks trimmed mt genome alignment is found as Electronic Supplementary Material.

## Supplementary information

supplementary information.

supplementary information 2.
