# Supplementary material for: Ancient DNA and high-resolution chronometry reveal a long-term human role in the historical diversity and biogeography of the Bahamian hutia
Source: Sci Rep. 2020 Jan 28;10:1373. doi: 10.1038/s41598-020-58224-y (PMC6987171; doi:10.1038/s41598-020-58224-y)
Supplement: Supplementary file 2 — supplementary information 2. [file 41598_2020_58224_MOESM2_ESM.pdf]

Supplementary Material for

Ancient DNA and high-resolution chronometry reveal a long-term human role in the historical diversity and biogeography of the Bahamian hutia

Jessica A. Oswald[1,2*], Julie M. Allen[2,3], Michelle J. LeFebvre[1], Brian J. Stucky[1], Ryan A. Folk[1,4], Nancy A. Albury[5], Gary S. Morgan[6], Robert P. Guralnick[1], David W. Steadman[1]

1. Florida Museum of Natural History, University of Florida, Gainesville, FL 32611
2. Biology Department, University of Nevada Reno, Reno, NV 89557
3. Illinois Natural History Survey, University of Illinois Urbana-Champaign, Urbana, IL 61801
4. Department of Biological Sciences, Mississippi State University, Starkville, MS 39762
5. National Museum of The Bahamas, Marsh Harbour, Abaco, The Bahamas.
6. New Mexico Museum of Natural History, 1801 Mountain Road NW, Albuquerque, NM 87104

*Correspondence: Jessica A. Oswald, 1659 Museum Road, Dickinson Hall, University of Florida, Gainesville, FL 32601. Email: oswaldj3@gmail.com.

This PDF file includes

Supplementary Tables 1-4

Supplementary Figures 1-4

Table S1. Radiocarbon (^{14}C) and stable isotope ($\delta^{13}C$, $\delta^{15}N$) data for individual bones of hutia (*Geocapromys ingrahami*) from the Bahamian Archipelago, proceeding roughly north-to-south in the island group. Beta-24481 is an old, time-averaged date determined on multiple bones (Morgan 1989). **Banks:** CAB, Crooked-Acklins Bank; CB, Caicos Bank; GBB, Great Bahama Bank; LBB, Little Bahama Bank; MB, Mayaguana Bank. **Site type:** (C), cultural site; (NC), non-cultural site. ^{14}C ages presented at 95.4% confidence (2σ). All analyses done at Beta Analytic Inc., Miami, FL. **, sample used successfully for aDNA extraction and is listed with associated UF FLMNH catalog number. *, sample used for aDNA extraction but did not yield a sequence. $\delta^{13}C$ and $\delta^{15}N$ are reported in per mille (o/oo)**.** In the ^{14}C age (cal BP) column "post-0" means that the sample retains enough ^{14}C in it that it cannot (with statistical certainty) be distinguished from 0 (set at AD 1950).

Bank	Island	Site	Skeletal element	Beta-number	Conventional ^{14}C age (yr BP)	$\delta^{13}C$ (o/oo)	^{14}C age (cal BP)	^{14}C age (cal BC/AD)	$\delta^{15}N$ (o/oo)
LBB	Abaco	Hole-in-the-Wall Cave (NC)	Ulna	411061	200 ± 30	-20.7	300-265, 220-140, 25-post 0	1650-1685, 1730-1810, 1925-post 1950 AD	+5.9
LBB	Abaco	Hole-in-the-Wall Cave (NC)	Femur	411060	280 ± 30	-19.2	430-355, 330-285	1520-1595, 1620-1665 AD	+7.2
LBB	Abaco	Hole-in-the-Wall Cave (NC)	**Femur (UF 322959)	520445	320 ± 30	-19.5	468-304	1482-1646 AD	+7.9
LBB	Abaco	Ralph's Cave (NC)	**Sacrum (UF 322961)	502523	420 ± 30	-21.5	524-435, 352-332	1426-1515, 1598-1618 AD	+3.9

GBB	Eleuthera	Garden Cave (NC)	Femur	330400	290 ± 30	-20.1	450-450, 440-350, 330-290	1500-1500, 1510-1600, 1620-1660 AD	+6.1
GBB	Eleuthera	Garden Cave (NC)	Ulna	338513	1390 ± 30	-19.9	1340-1280	610-670 AD	+7.1
GBB	Eleuthera	Garden Cave (NC)	**Sacrum (UF 322948)	520443	2000 ± 30	-19.8	2033-2029, 2004-1880	84-80 BC, 55 BC – 70 AD	+6.3
GBB	Eleuthera	Garden Cave (NC)	Femur	330401	2180 ± 30	-19.1	2350-2300, 2240-2180	400-350, 290-230 BC	+6.9
GBB	Eleuthera	Garden Cave (NC)	Femur	330404	3980 ± 30	-18.9	4520-4460, 4450-4410	2570-2510, 2550-2460 BC	+7.5
GBB	Eleuthera	Garden Cave (NC)	Humerus	330403	4270 ± 30	-19.3	4860-4830	2910-2880 BC	+7.0
GBB	Eleuthera	Garden Cave (NC)	**Tibia (UF 322947)	520442	5270 ± 30	-19.5	6180-6144, 6125-5939	4231-4195, 4176-3990 BC	+7.3
GBB	New Providence	Banana Hole (NC)	Various bones	24481	7980 ± 230	---	9432-8392	---	---
GBB	New Providence	Banana Hole (NC)	Femur	432277	No collagen	---	---	---	---
GBB	New Providence	Banana Hole (NC)	Femur	432278	No collagen	---	---	---	---

GBB	Long Island	Hanging Garden Crawl Through Cave	Femur	494888	No collagen	---	---	---	---
GBB	Long Island	Hanging Garden Crawl Through Cave	Femur	494889	No collagen	---	---	---	---
GBB	Long Island	Hanging Garden Crawl Through Cave	Femur	494890	No collagen	---	---	---	---
GBB	Long Island	Hanging Garden Crawl Through Cave	Femur	494891	No collagen	---	---	---	---
GBB	Long Island	Hanging Garden Crawl Through Cave	Femur	494892	No collagen	---	---	---	---
GBB	Long Island	Hanging Garden Crawl Through Cave	Femur	494893	No collagen	---	---	---	---
CAB	Crooked	Crossbed Cave	Ulna	411055	330 ± 30	-20.1	485-305	1465-1645 AD	+6.9
CAB	Crooked	McKay's Bluff Cave (NC)	Tibia	411057	350 ± 30	-20.7	500-310	1450-1640 AD	+5.1
CAB	Crooked	McKay's Bluff Cave (NC)	Femur	411059	380 ± 30	-20.7	505-425, 395-320	1445-1525, 1555-1630 AD	+4.7
CAB	Crooked	McKay's Bluff Cave (NC)	**Femur (UF 322960)	502524	380 ± 30	-20.6	505-426, 392-318	1445-1524, 1558-1632 AD	+1.5

CAB	Crooked	McKay's Bluff Cave (NC)	Ulna	411058	400 ± 30	-19.1	510-430, 355-330	1440-1520, 1595-1620 AD	+5.2
CAB	Crooked	McKay's Bluff Cave (NC)	**Pelvis (UF 322949)	520444	420 ± 30	-20.3	524-434, 353-332	1426-1516, 1597-1618 AD	+7.5
MB	Mayaguana	Abraham's Bay Cave 2 (NC)	*Humerus	520447	200 ± 30	-20.4	304-260, 222-140, 25 – post 0	1646-1690, 1728-1810, 1925 – post 1950 AD	+13.2
MB	Mayaguana	Pond Apple Cave (NC)	*Humerus	520448	280 ± 30	-19.9	452-448, 438-350, 334-284, 166-155	1498-1502, 1512-1600, 1616-1666, 1784-1795 AD	+9.1
CB	Providenciales	Palmetto Junction (C)	*Femur	520446	530 ± 30	-19.8	630-600, 560-510	1320-1350, 1390-1440 AD	+6.6

Table S2. Pairwise distance of *Geocapromys* samples based on the GBlocks [59] trimmed dataset. KU892768.1 *Geocapromys ingrahami* is the modern sample from East Plana Cay.

	UF 322961 *G. ingrahami* Abaco shotgun	UF 322959 *G. ingrahami* Abaco enrichment	UF 322948 *G. ingrahami* Eleuthera shotgun	UF 322947 *G. ingrahami* Eleuthera enrichment	UF 322960 *G. ingrahami* Crooked Island shotgun	UF 322949 *G. ingrahami* Crooked Island enrichment	KU892768.1 *Geocapromys ingrahami*	KU892767.1 *Geocapromys brownii*
UF 322961 *G. ingrahami* Abaco shotgun	0.000	0.000	0.002	0.003	0.020	0.021	0.024	0.046
UF 322959 *G. ingrahami Abaco* enrichment	0.000	0.000	0.002	0.003	0.020	0.021	0.024	0.046
UF 322948 *G. ingrahami* Eleuthera shotgun	0.002	0.002	0.000	0.003	0.020	0.021	0.023	0.046
UF 322947 *G. ingrahami* Eleuthera enrichment	0.003	0.003	0.003	0.000	0.020	0.020	0.023	0.046
UF 322960 *G. ingrahami* Crooked Island shotgun	0.020	0.020	0.020	0.020	0.000	0.006	0.008	0.045

UF 322949 *G. ingrahami* Crooked Island enrichment	0.021	0.021	0.021	0.020	0.006	0.000	0.004	0.046
KU892768.1 *Geocapromys ingrahami*	0.024	0.024	0.023	0.023	0.008	0.004	0.000	0.048
KU892767.1 *Geocapromys brownii*	0.046	0.046	0.046	0.046	0.045	0.046	0.048	0.000

Table S3. Samples from GenBank that were included in phylogenetic analyses.

KU892766.1	*Capromys*	*pilorides*
KU892752.1	*Carterodon*	*sulcidens*
JX312692.1	*Chinchilla*	*lanigera*
KU892753.1	*Clyomys*	*laticeps*
NC20659.1	*Ctenomys*	*leucodon*
KU762015.1	*Dactylomys*	*dactylinus*
KU892781.1	*Echimys*	*chrysurus*
KU892755.1	*Euryzygomatomys*	*spinosus*
KU892767.1	*Geocapromys*	*brownii*
KU892768.1	*Geocapromys*	*ingrahami*
KU892779.1	*Hoplomys*	*gymnurus*
KU892785.1	*Isothrix*	*sinnamariensis*
KU892775.1	*Kannabateomys*	*amblyonyx*
KU892786.1	*Lonchothrix*	*emiliae*
KU892782.1	*Makalata*	*didelphoides*
NC37779.1	*Makalata*	*macrura*
KU892787.1	*Mesomys*	*hispidus*
KU892788.1	*Mesomys*	*stimulax*
MH182628.1	*Myocastor*	*coypus*
KU892770.1	*Mysateles*	*prehensilis*
HM544134.1	*Octodon*	*degus*
KU892756.1	*Phyllomys*	*blainvillii*
KU892771.1	*Plagiodontia*	*aedium*
NC39099.1	*Proechimys*	*cuvieri*
KX688206.1	*Proechimys*	*longicaudatus*
NC39370.1	*Proechimys*	*quadruplicatus*
NC39444.1	*Proechimys*	*simonsi*
HM544133.1	*Spalacopus*	*cyanus*
KU892773.1	*Thrichomys*	*apereoides*
KU892761.1	*Trinomys*	*albispinus*
JX312694.1	*Trinomys*	dimidiatus
KU892762.1	*Trinomys*	*iheringi*
KX650080.1	*Trinomys*	*moojeni*
KU892763.1	*Trinomys*	*paratus*
KX655539.1	*Trinomys*	*setosus*
KU892765.1	*Trinomys*	*yonenagae*
HM544132.1	*Tympanoctomys*	*barrerae*

Table S4. jModelTest [60, 61] output for the mt genome dataset.

Model	-lnL	K	AIC	Delta	Weight	Cumulative Weight
GTR+I+G	195901.58	94	391991.15	0	1	1
TPM1uf+I+G	195952.49	91	392086.99	95.84	1.55e-021	1
TrN+I+G	195978.83	81	392139.66	148.51	5.63e-033	1

Figure S1. MapDamage [58] plots for each of the ancient *Geocapromys ingrahami* samples included in our analyses organized by Bahamian island.

A. Fragment misincorporation plots for samples from Abaco. Left plot is the shotgun sequenced data (UF 322961), right plot is the targeted enrichment sample (UF 322959).

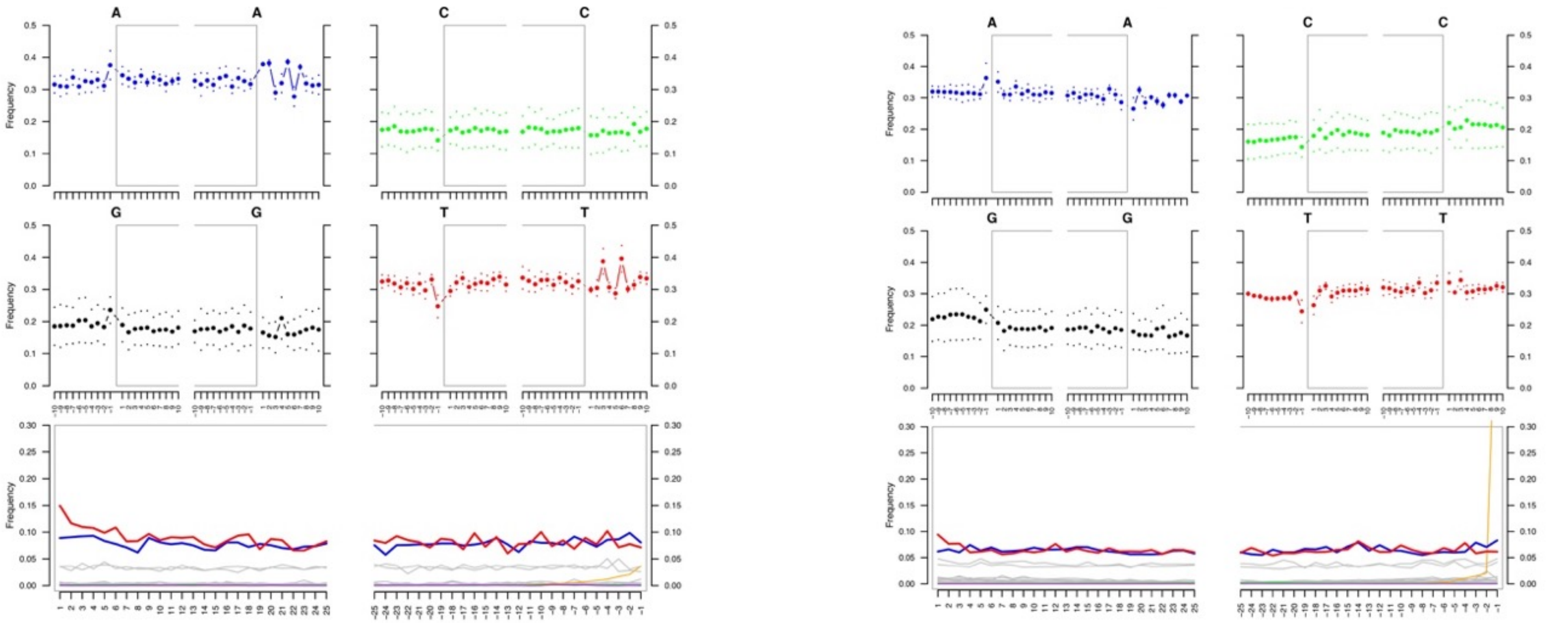

B. Fragment misincorporation plots for samples from Eleuthera. Left plot is the shotgun sequenced data (UF 322948), right plot is the targeted enrichment sample (UF 322947).

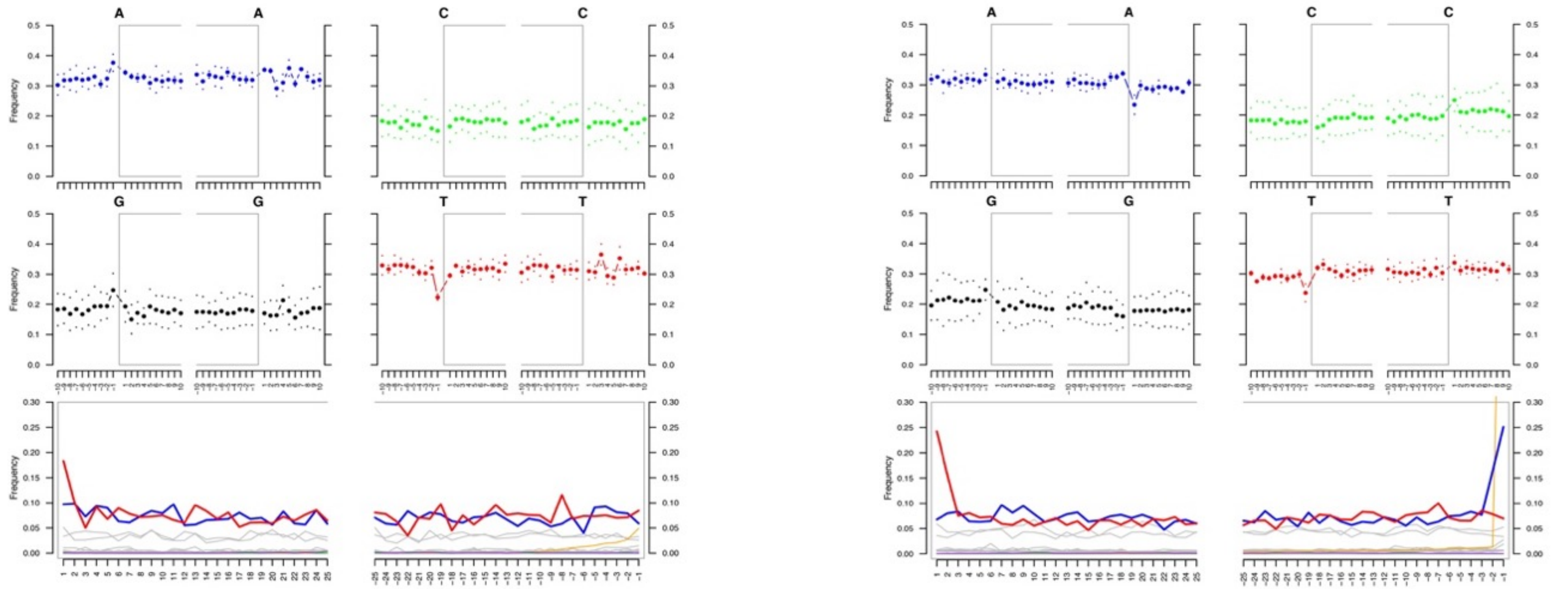

C. Fragment misincorporation plots for samples from Crooked Island. Left plot is the shotgun sequenced data (UF 322960), right plot is the targeted enrichment sample (UF 322949).

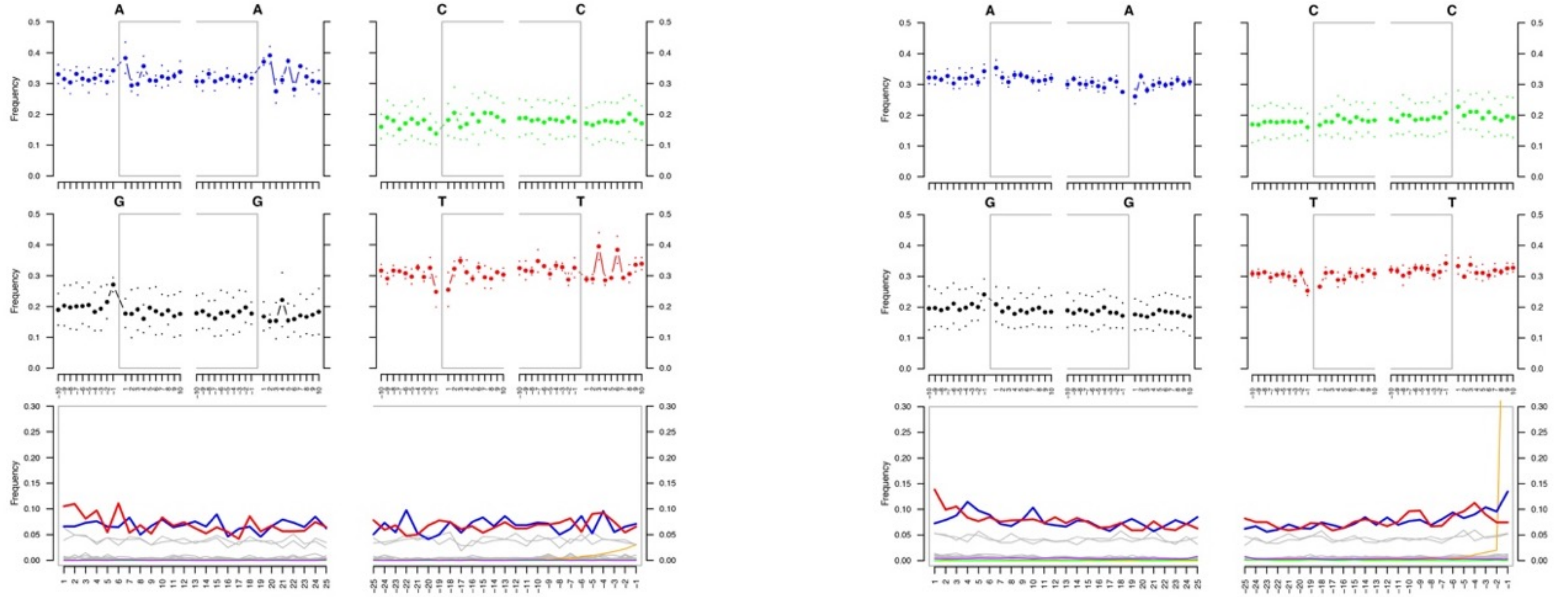

Figure S2. Partitioned RAxML [62] phylogeny. Bootstrap support is on the nodes. The *Geocapromys ingrahami samples* used for this study have the island they were sampled from in the label name. Shotgun sequenced samples have a "1" in the label, target enriched samples have a "2" in the label. LBB, Little Bahama Bank; GBB, Great Bahama Bank; CAB, Crooked-Acklins Bank.

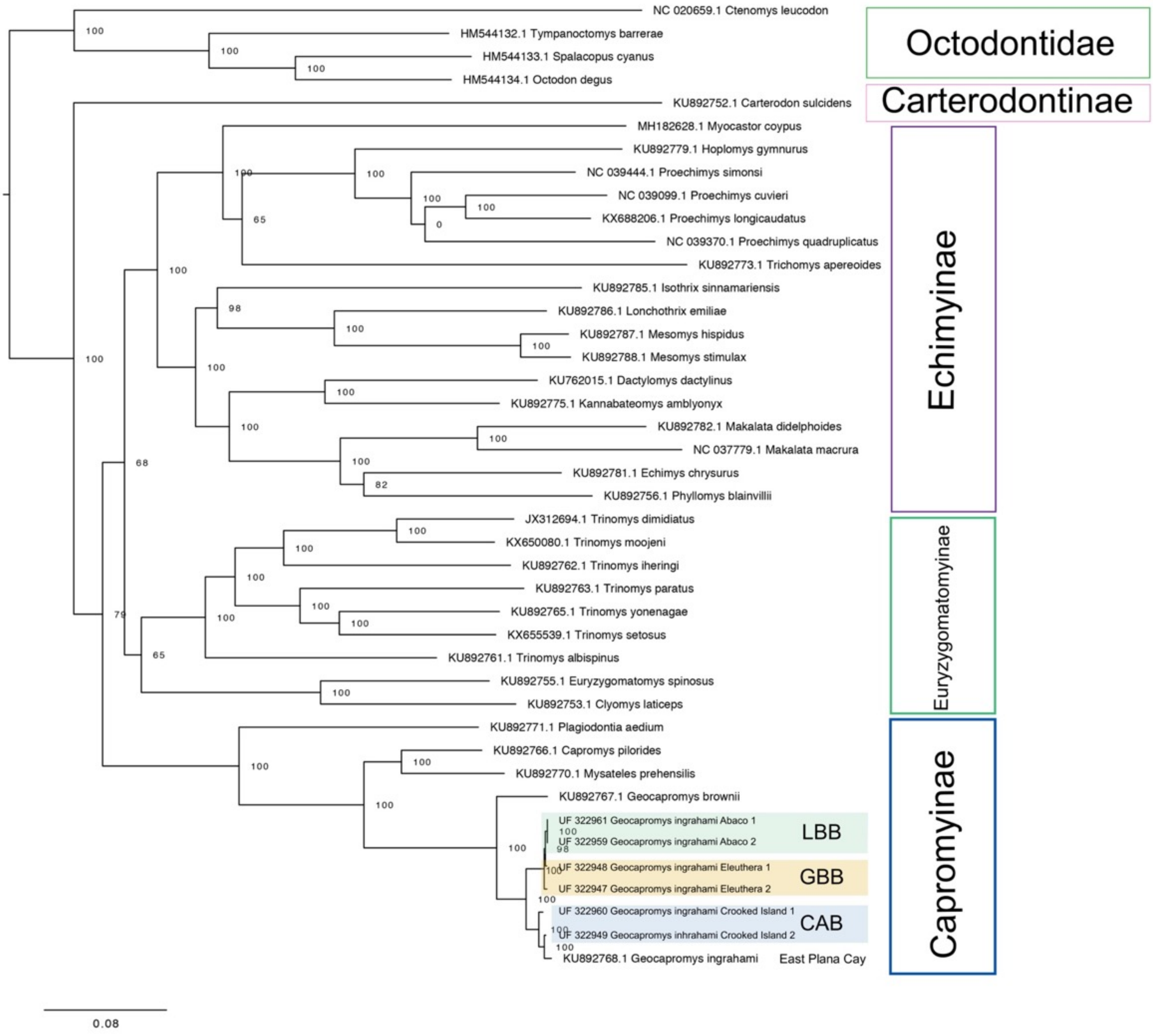

Figure S3. BEAST2 [63] phylogeny with the 95% highest posterior density divergence time represented in millions of years on the nodes in parentheses followed by the median divergence time. The median divergence time for *Geocapromys ingrahami* is represented in Figure 3. The BEAST2 [63] phylogeny presented in the main text is a cropped version of this phylogeny. The Miocene Epoch (23 – 5.3 mya) is indicated by the purple box. The Pliocene Epoch (5.3 – 2.6 mya) is indicated by the white box. The Pleistocene Epoch 2.6 Ma to ~12,000 years ago is light blue. The Holocene Epoch began at the end of Pleistocene.

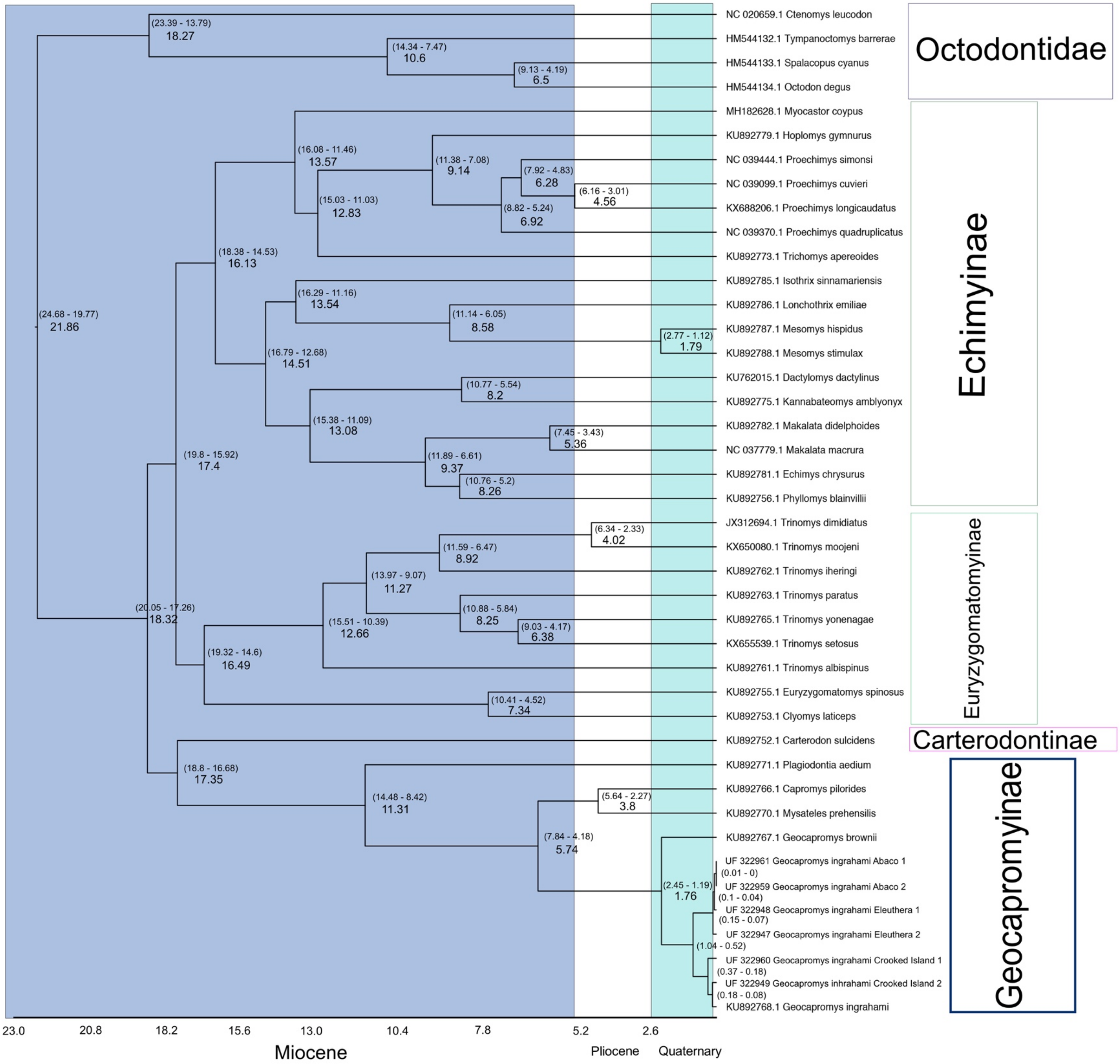

Figure S4. BEAST2 [63] phylogeny with posterior probability on the nodes. The color scheme for geological epochs is the same as Fig. S3.

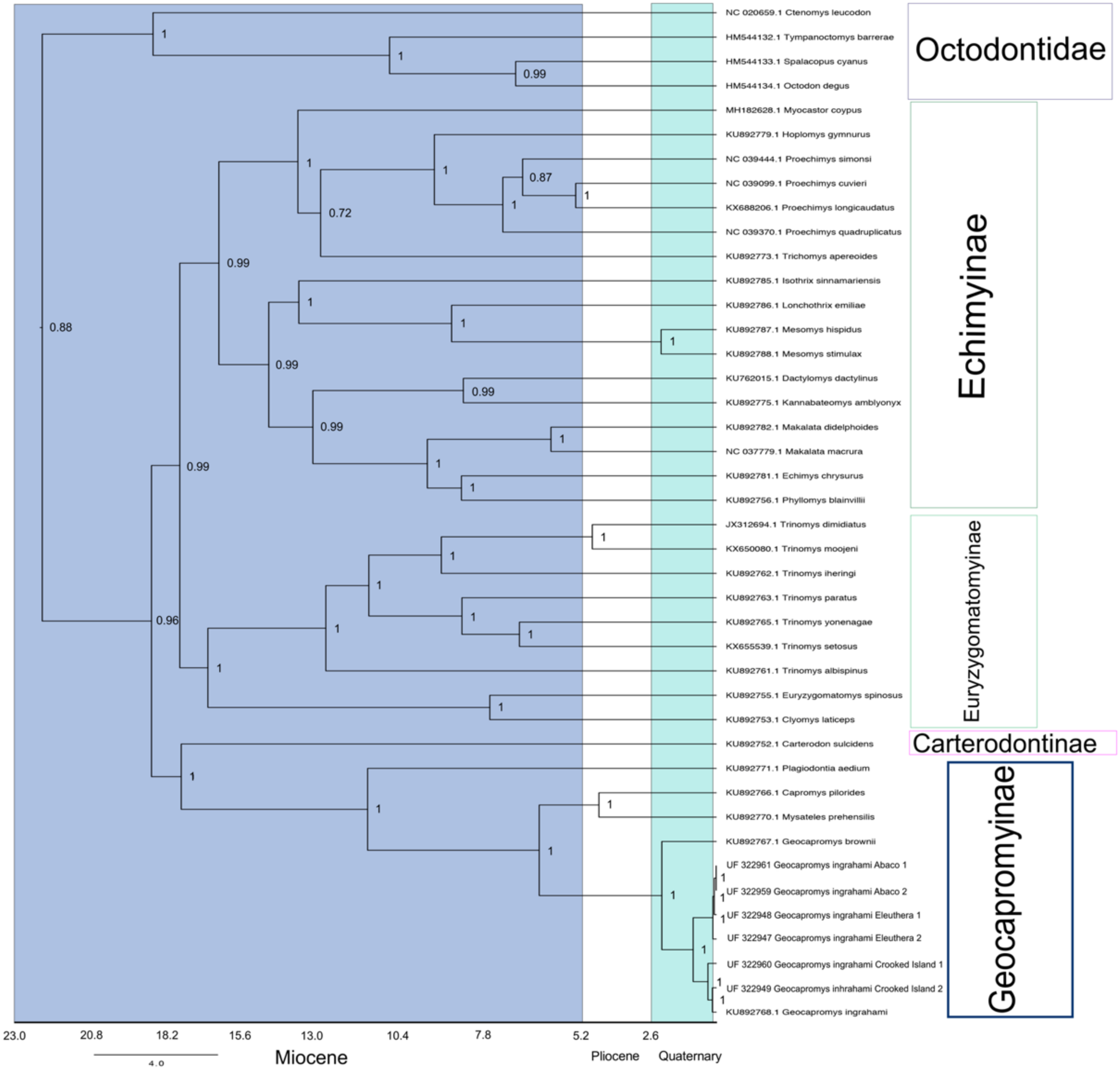

Supplementary Material References. (The numbering matches first mention in the main text.)

58. Jónsson, H., Ginolhac, A., Schubert, M., Johnson, P. L. F. & Orlando, L. mapDamage2.0: fast approximate Bayesian estimates of ancient DNA damage parameters. *Bioinformatics* **29**, 1682–1684 (2013).

59. Castresana J. Selection of conserved blocks from multiple alignments for their use in phylogenetic analysis. *Mol. Biol. Evol.* **17**, 540-552 (2000).

60. Guindon, S. & Gascuel, O. A simple, fast and accurate method to estimate large phylogenies by maximum-likelihood. *Syst. Biol.* **52**, 696-704 (2003).

61. Darriba, D., Taboada, G. L., Doallo, R. & Posada, D. jModelTest 2: more models, new heuristics and parallel computing. *Nat. Methods* **9**, 772 (2012).

62. Stamatakis, A. RAxML Version 8: A tool for phylogenetic analysis and post-analysis of large phylogenies. *Bioinformatics* **30**, 1312-3 (2014).

63. Bouckaert, R. *et al*. BEAST 2.5: An advanced software platform for Bayesian evolutionary analysis. *PLOS Comput. Biol.* **15**, e1006650 (2019).
